# The contribution of type-I IFN-mediated neuroinflammation to Parkinson's disease progression

**DOI:** 10.1016/j.bbih.2025.101017

**Published:** 2025-05-21

**Authors:** Shuyan Chen, Peter J. Crack, Juliet M. Taylor

**Affiliations:** Neuropharmacology Laboratory, Department of Biochemistry and Pharmacology, University of Melbourne, Parkville, Australia

**Keywords:** Neuroinflammation, Aging, Type-I interferons, Parkinson's disease, Microglia

## Abstract

Parkinson's disease (PD) is a chronic neurodegenerative disease characterized by motor dysfunction. Pathological hallmarks of the disease include selective dopaminergic neuronal death, intraneuronal deposits known as Lewy bodies and extensive neuroinflammation within the central nervous system (CNS). Microglia are the key cellular players in mediating this neuroinflammatory response, propagating this neuropathology to exacerbate the neuronal cell death. Growing evidence suggests a role for the type-I interferons (IFN) in driving the neuroinflammatory response in PD, with increased type-I IFN signatures reported in both PD patients and in animal models of the disease. This review will discuss 1) the key players that modulate the neuroinflammatory response in PD and their implications in the CNS 2) the contribution of the type-I IFNs in driving the neuroinflammatory response in PD, and 3) evidence for therapeutically targeting type-I IFN signalling to slow disease progression. A greater understanding of the underlying mechanisms that lead to the elevated neuroinflammatory response in PD could lead to new advances in therapeutic targets that effectively slow the disease progression.

## Highlights

• Summarises how type-I interferon signalling contributes to Parkinson’s disease progression via innate immune activation.
• Describes how type-I interferons and microglia drive chronic neuroinflammation and pathology in Parkinson’s disease.
• Reviews neuroinflammation in Parkinson’s disease with emphasis on the role of type-I interferons in CNS immune dysregulation.

## 1 Introduction

Parkinson's disease (PD) is the second most common neurodegenerative disease worldwide, affecting 1 % of the population over 60 years old (Dorsey and Bloem, 2018; de Lau and Breteler, 2006). To date, the precise etiology of PD remains unknown with all current therapies failing to address the progressive nature of the disease. Pathologically, PD is characterized by a progressive loss of dopaminergic (DA) neurons, primarily in the substantia nigra pars compacta (SNpc), with the surviving DA neurons displaying intracytoplasmic inclusion bodies, known as Lewy bodies (LB) (Rocha et al., 2018). A major component of LB is α-synuclein (α-syn), with this protein implicated in the disease progression through a number of mechanisms, including the initiation of inflammatory events within the central nervous system (CNS) (Colla et al., 2012; Barrett and Timothy Greenamyre, 2015). Importantly, growing evidence supports that, in PD, there is an chronic neuroinflammatory response, initiated through an unknown trigger, that contributes to the progression and exacerbation of the disease (Mogi et al., 1996; Kempuraj et al., 2017; Gordon et al., 2018). Such dysregulation results in a positive feedback loop that reinforces the detrimental neuroinflammatory cascade.

This review will discuss the complexity of the neuroinflammatory response within the CNS in a healthy and PD setting. Specifically, the contribution of specific cellular players in this response will be highlighted with a focus on the role of the type-I interferons in driving the self-perpetuating neuroinflammation in neurodegenerative diseases including PD. This review summarises evidence supporting that type-I interferons may be a promising therapeutic target to slow PD progression.

## 2 Neuroinflammation: an overview

Neuroinflammation refers to the coordinated immune response within the CNS triggered by pathological insults. Neuroinflammation acts as a defense mechanism to promote tissue repair, clear cellular debris, and restore homeostasis (Baufeld et al., 2018). The process is marked by the production of various pro-inflammatory mediators including Interleukin-1β (IL-1β), Interleukin-6 (IL-6) and Tumor Necrosis Factor α (TNF-α) primarily from reactive microglia, which further recruits additional immune cells such as astrocytes to the injury site to promote repair (Minter et al., 2016a). In addition to microglia that form the first line of defense, neurons can also contribute to the local inflammatory response by generating and expressing inflammatory molecules, which may ultimately elevate oxidative stress and cause damage to themselves (Heneka et al., 2010). Under pathological conditions, this is compounded by peripheral immune cells and pro-inflammatory mediators secreted that have gained entry into the CNS through a compromised blood-brain barrier (BBB) (Capuron and Miller, 2011; Joers et al., 2017). Although initially beneficial, an elevated neuroinflammatory process that remains chronically unresolved can pose a concerning risk to the cellular environment.

## 3 Microglia

Microglia are the principal immune cells of the CNS and key players in the neuroinflammatory cascade. Microglia survey the microenvironment, provide trophic support to the CNS and initiate the pro-inflammatory response following detection of cellular damage signals (Rocha et al., 2018; Leng and Edison, 2021). Under physiological conditions, resting microglia present as ramified cells with long processes, that maintain contact with neurons, astrocytes, and blood vessels, whereas activated microglia adopt an amoeboid shape with enlarged cell bodies and shorter processes (Colonna and Butovsky, 2017). The activated state of microglia is characterized by self-proliferation, recruitment of monocytes, upregulated expression of major histocompatibility complex II (MHC-II), and the release of pro-inflammatory cytokines including but not limited to TNF-α, IL-1β, and IL-6 (Lagarde et al., 2018; Venneti et al., 2009). Among these pro-inflammatory cytokines, it has been reported that microglia induce the production of different inflammatory cytokines through altering the combinations of MAPKs and the presence of NO to achieve differential response (Ishijima and Nakajima, 2021). TNF-α has also been shown to directly bind to TNF receptors on the dopaminergic neurons in the midbrain and initiate the pro-apoptotic cellular process (L'Episcopo et al., 2018). Moreover, the enhanced antigen-presenting capacities to T cells allows microglia to synchronize the communication between innate and adaptive immune systems (Shaked et al., 2004). The ability of modulating key cellular responses, together with their own robust production of pro-inflammatory cytokines, make microglia a critical mediator of neuroinflammation within the CNS (Colonna and Butovsky, 2017).

As an essential class of glial supporting cells, microglia maintain close contact with neurons. Microglia migrate into the CNS early in prenatal development and play important roles in guiding neurons to structure and connect, in shaping neuronal synapses, as well as in regulating synaptic pruning and plasticity (Vezzani and Viviani, 2015; Paolicelli et al., 2011). When neurons are exposed to harmful stimuli, damaged neurons release signals to the surrounding microglia and prompt them to become activated and initiate neuroinflammatory processes. Mediators such as CX3C chemokine, fractalkine, and interleukin-34 have been implicated in this activation (Suzumura, 2013). Critically, it has been shown that enhanced neuronal activity can result in neurogenic neuroinflammation, a concerted response involving not only microglia but also astrocytes, neurons, and vascular cells (Xanthos and Sandkuhler, 2014).

## 4 Astrocytes and other immune cells

Astrocytes are the most abundant glial cell type in the CNS and are crucial in the maintenance of brain homeostasis (Sofroniew and Vinters, 2010). Specifically, they are involved in the regulation of cerebral blood flow, the maintenance of synaptic homeostasis and neurotropic support (Leng and Edison, 2021). Importantly, astrocytes are active players in neuroinflammation as they are a part of the microglial-astrocytic crosstalk that is fundamental to CNS health. Numerous signalling molecules have been identified as key mediators of this communication. Microglial IL-1β promotes the expression of tissue inhibitor of matrix metalloproteinases in astrocytes (Welser-Alves et al., 2011; Hewett et al., 1993), whilst astrocytic GDNF modulates microglial activation and phagocytosis (Rocha et al., 2012). Microglia are rapidly attracted to the site of injury through the action of ATP, which is released and sustained by the surrounding astrocytes, enabling efficient microglia recruitment and proper repair (Davalos et al., 2005). In addition, activated microglia signal astrocytes to activate signalling pathways including NFκB, STATs, and mitogen-activated protein kinase (MAPK) pathways, which in turn release more pro-inflammatory cytokines, promote changes in the permeability of the BBB to allow peripheral immune cell infiltration, and together result in an amplified immune response (Jha et al., 2019).

## 5 Neuroinflammation in Parkinson's disease

Neuroinflammation has become an increasing focus of PD research with inflammatory mediators shown to play a role in exacerbating the pathology and degeneration of DA neurons. On the basis of distinct disease stages observed in PD patients, the Braak hypothesis was proposed to explain the progression of the disease by correlating symptoms with specific brain regions being affected by the abnormal α-syn pathology (Braak et al., 2003a). Indeed, the spatial and temporal progression sequence of PD remains largely unknown, however is believed to be related to the interrelationships between misfolded α-syn and neuroinflammation (Tansey and Goldberg, 2010; Taylor et al., 2018; Olanow et al., 2019). Specifically, a positron emission tomography (PET) imaging study revealed extensive microglial activation in PD patient brains, suggesting an active role for microglia in the disease pathogenesis (Edison et al., 2013; Terada et al., 2016). In addition, the substantia nigra pars compacta (SNpc) is a region that is densely populated by microglia, predisposing it to a regional vulnerability to oxidative stress and a potentiating, pro-inflammatory drive caused by active microglia (Lawson et al., 1990).

**Fig. 1:**
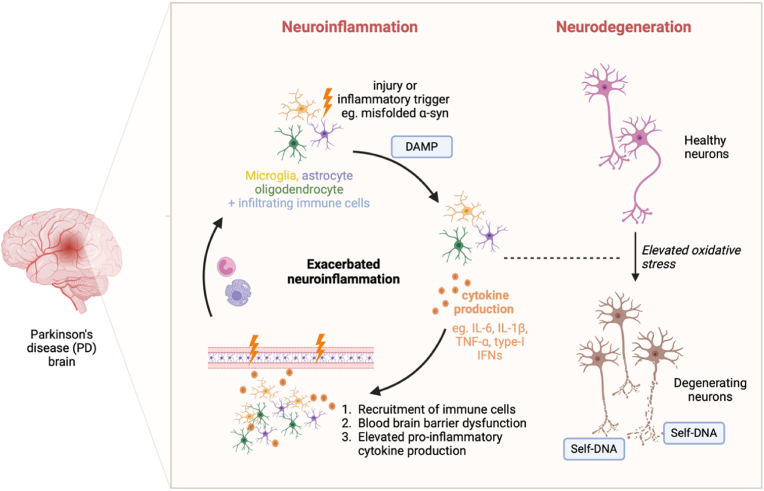
**Neuroinflammatory cascade in PD.** The release of damage signals in response to an injury or inflammatory trigger, leads to the activation of microglia. This leads to the secretion of pro-inflammatory cytokines including TNF-α, IL-1β, IL-6, and type-I IFNs, to the initiation of local pro-inflammatory changes. The chronic presence of inflammatory mediators and excessive population of immune cells in the microenvironment creates ongoing stress to the neurons, including mitochondrial dysfunction. This leads to the degeneration of neurons which release self-DNA that further activates microglia to combat ‘cellular damage’ with a neuroinflammatory response. This positive feedback loop ultimately leads to the accelerated disease progression. α-syn: α-synuclein. DAMP: Damage associated molecular pattern.

A genetic link between microglial activation and neuroinflammation has also been reported in PD. A genome-wide association study (GWAS) identified a single nucleotide polymorphism (SNP) in the human leukocyte antigen (*HLA-DRA*) gene as a genetic risk factor for PD (Hamza et al., 2010). The gene encodes the HLA-DR antigens present on antigen-presenting cells including microglia, implying the involvement of the immune system as well as microglial activation in PD pathogenesis (Joers et al., 2017). In post-mortem PD brains, reactive microglia have been identified in the SN and striatum, the same regions which display increased levels of pro-inflammatory cytokines were reported, including IL-1β, IL-6, and TNF-α (De Virgilio et al., 2016; Mogi et al., 1994; Muller et al., 1998; Imamura et al., 2003). With evidence supporting a specific role for microglia in mediating α-syn transport, it has been suggested that when microglia actively participate in an inflammatory response, the efficiency of α-syn clearance becomes compromised (Lee et al., 2008; George and Brundin, 2015). Thus, it could be speculated that, in PD, with the extensive activation of microglia orchestrating neuroinflammation, α-syn accumulates and misfolds, stimulating a robust inflammatory response that ultimately results in neurotoxicity (Fig. 1).

Several anti-inflammatory approaches have shown beneficial effects in rescuing PD phenotypes in animal models of the disease. The microglial inhibitor minocycline has displayed neuroprotective effects by preventing nigral dopaminergic neurodegeneration (Du et al., 2001; Wu et al., 2002). The administration of IL-10, an anti-inflammatory cytokine, showed significant protection against dopaminergic neuron loss and behavioural deficits in a rat model (Johnston et al., 2008; Qian et al., 2006). Moreover, the inhibition of NFκB signalling suppressed microglial activation and prevented dopaminergic neuronal loss in a mouse model of PD (Ghosh et al., 2007; Xing et al., 2007). However, a greater understanding of the over-arching pathological processes, their contribution to neuroinflammation and how it potentiates nigral degeneration in PD is needed to validate these potential therapeutic targets.

One theory that potentially explains the beneficial effects seen with anti-inflammatory approaches is that chronic neuroinflammation results in elevated oxidative stress, which in time induces cellular damage in PD. Oxidative stress is the result of dysregulated cellular redox activity where the production of reactive oxygen species would surpass the maintenance capacity of endogenous antioxidant enzymes and molecular chaperones (Trist et al., 2019). The accumulation of these reactive oxygen species such as superoxide, hydrogen peroxide and lipid hydroperoxides can cause oxidative damage that compromises neuronal integrity and function (Schieber and Chandel, 2014). Importantly, elevated oxidative stress is shown to be a robust pathological feature in early-stage PD, likely preceding dopaminergic neuronal loss and accompanying the early neuroinflammatory changes in PD brains (Trist et al., 2019; Ferrer et al., 2011). Accumulating evidence from numerous animal models of PD suggests that oxidative stress could be a causative factor. It was reported that paraquat resulted in enhanced nitric oxide synthase expression and lipid peroxidation in mice, confirming a heightened state of induced oxidative stress following the herbicide exposure. Importantly, treatment with the herbal plant Mucuna pruriens was able to attenuate the nitric oxide production and was correlated with improved survival of dopaminergic neurons in the SN (Yadav et al., 2017). Similarly, the PD-related neurotoxin MPTP also induces neurotoxicity through oxidative stress. It was shown that ursolic acid, known for its anti-inflammatory and antioxidant activity, improved behavioural deficits and rescued dopaminergic neurons in MPTP-treated mice (Rai et al., 2016). Indeed, nigral dopaminergic neurons have been suggested to be vulnerable against oxidative stress, likely due to the dense population of microglia residing in the region and the resultant priming of the pro-inflammatory environment. Moreover, aging is believed to further compromise neuronal health by imposing an age-dependent decline in the synaptic firing and conductance of dopaminergic neurons (Rai et al., 2016; Branch et al., 2016). Thus, the selective vulnerability of these neurons in PD could be attributed to and driven by the heightened oxidative burden mediated by microglia, that is known to be worsened as we age.

In summary, microglia are key contributors to the self-perpetuating neuroinflammatory response in the PD brain with α-syn accumulation resulting from inefficient clearance the initiating trigger (Whitton, 2007). Microglia, together with other cellular players, are primed to sustain these pro-inflammatory changes, leading to a deleterious chain of events. Within the neuroinflammatory cascade, the type-I interferons (IFNs) have been implicated as key mediators of this self-perpetuating response in PD.

## 6 Type-I Interferon signalling pathway

**Fig. 2:**
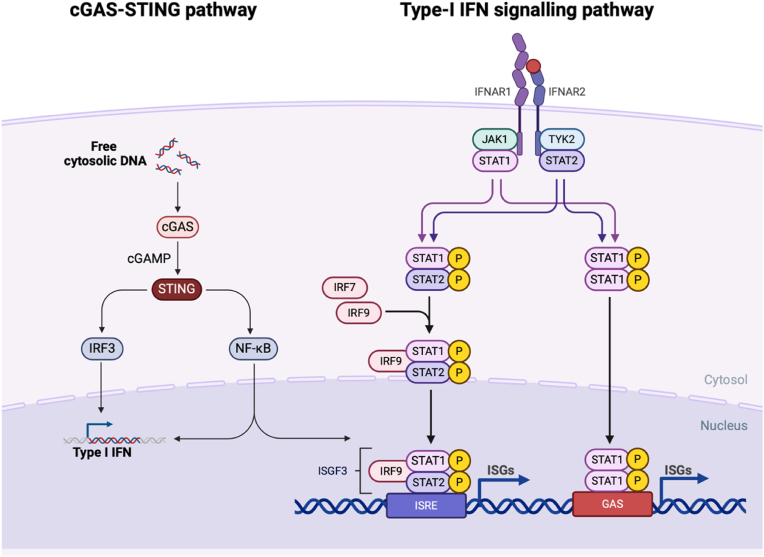
**Type- I IFN signalling pathway** IFNα/β binding activates the type-I IFN signalling pathway via receptor dimerization which subsequently recruits and phosphorylates JAK1 and TYK2, which in turn activate the STAT proteins including STAT1/2/3 and IRF transcription factors such as IRF3/7/9. The phosphorylated IRF/STAT complex enters the nucleus where it binds to response elements on DNA to upregulate gene transcription of pro-inflammatory mediators including TNF-α, IL-1β, IL-6, and interferon-regulated genes. cGAS-STING: cyclic GMP-AMP synthase-stimulator of interferon genes. IFN: Interferon. IRF: Interferon regulatory factor. ISRE: Interferon stimulated response element. JAK-STAT: Janus-associated kinase and signal transducer and activator of transcription.

IFNs are a family of pleiotropic cytokines, originally identified by their ability to ‘interfere’ with viral replication. Three types of IFN have been classified, with type-I and type-II being responsible for regulating and activating the immune response (Parkin and Cohen, 2001). Type-I IFNs signal through their cognate receptor, the type-I interferon receptor alpha-1, IFNAR, consisting of IFNAR1 and IFNAR2 subunits (de Weerd and Nguyen, 2012; Isaacs and Lindenmann, 1957; de Weerd et al., 2007) (Fig. 2). The IFNAR receptor dimerization will activate the Janus-associated kinase and signal transducer and activator of transcription (JAK-STAT) pathway, followed by phosphorylation of Interferon Regulatory Factors (IRFs) and STAT proteins. These transcription factors modulate gene transcription of a number of classical pro-inflammatory genes, including type-I IFNs themselves (Schindler et al., 2007). Alternatively, stimulator of interferon genes (STING), an endoplasmic reticulum (ER) protein can induce type-I IFN expression via an IRF-3 dependent pathway (Ishikawa and Barber, 2008). Collectively, overactivation of the type-I IFN pathway is believed to drive a chronic and self-sustained inflammation in the pathological context, elevating oxidative stress that will eventually become cytotoxic (de Weerd et al., 2007; Pestka et al., 2004).

## 7 Type-I IFNs: friend, foe or both?

Type-I IFNs are known to be key mediators of the inflammatory response and actively modulate numerous physiological processes. In response to viral infection, type-I IFNs engage with both innate and adaptive immunity by influencing the cytokine profile of T lymphocytes and by priming adaptive T cell responses (Huber and Farrar, 2011; Durbin et al., 2000). Studies investigating the constitutive expression of IFN-β in the absence of infection have demonstrated a homeostatic role for the cytokine and suggested that it is essential for maintaining stem cell niche, immune cell function, and bone remodeling (Gough et al., 2012; Tovey et al., 1987). Although type-I IFN signalling is relatively well-characterized in the periphery, its role in the CNS is less clear.

Type-I IFNs are currently used clinically for treating multiple sclerosis (MS). MS is an autoimmune, neurodegenerative disease that is characterized by demyelination of the nerves and chronic inflammation (Mahad et al., 2015). IFN-β is not only the first therapy approved for MS, but also remains the first-line disease-modifying treatment for the condition (Rommer and Zettl, 2018). Despite the effectiveness of IFN-β in reducing the severity of symptoms in MS, not all patients respond favourably and in some cases patients cease treatment due to ineffectiveness or side effects, including PD-like symptoms (Manouchehrinia and Constantinescu, 2012). Similarly, patients with other unrelated conditions have reported parkinsonism-like symptoms after receiving IFN-α treatment (Almeida et al., 2009; Kajihara et al., 2010). Thus, it is likely that type-I IFN signalling may have both homeostatic and pathogenic actions in the CNS.

Increased type-I IFN signalling has been implicated in the aging brain. Specifically, an elevated type-I IFN gene signature has been identified in the choroid plexus of both aged humans and mice. The negative impact of the elevated type-I IFN response was supported by the partial restoration of cognitive function in aged mice that received a blocking monoclonal antibody to the type-I IFN receptor, IFNAR1 (Baruch et al., 2014). More recently, a link between the type-I IFNs and microglial function in the aged brain has been reported. Microglia in the aged brain adopted a type-I IFN mediated pro-inflammatory phenotype that was associated with declining cognitive function (Deczkowska et al., 2017). It could be speculated that the low but sustained inflammation that occurs as we age provides a highly reactive and vulnerable niche for certain neurodegenerative diseases, including PD. Thus, when challenged with a secondary insult, for instance, a damage signal induced by intracellular α-syn aggregates, an aging brain responds with an amplified inflammatory response (Sierra et al., 2007; Yao and Zhao, 2018). In support of this, studies have shown that aged mice display exacerbated neuroinflammatory responses, denser population of activated microglia, and more profound and rapid loss of DA neurons when injected with PD-related toxins (Yao and Zhao, 2018; Buchanan et al., 2008; Munoz-Manchado et al., 2016). As aging is the greatest risk factor for PD, the elevated type-I IFN response may underlie the accelerated progression of PD pathologies in aged individuals.

Accumulating evidence supports that type-I IFNs are key drivers for the pro-inflammatory shift in the CNS and contribute to age-related CNS pathologies (Taylor et al., 2018; Hui et al., 2023). In AD post-mortem patients, the type-I IFNs, IFN-α and IFN-β are upregulated compared to age-matched controls (Taylor et al., 2014). In an animal model of AD, the APP_SWE_/PS1_ΔE9_ mouse, mice with ablated type-I IFN signalling (APP_SWE_/PS1_ΔE9_ x IFNAR1^−/−^), display reduced Aβ monomer levels, attenuated microgliosis and levels of pro-inflammatory cytokines that correlates with improved spatial learning and memory performance (Minter et al., 2016b). This has been supported by *in vitro* studies, with decreased levels of IL-1β, IL-6, and TNF-α reported in IFNAR1^−/−^ microglia treated with monomeric Aβ_1-42_ (Moore et al., 2020). The IFNAR1^−/−^ microglia were also observed with a greater phagocytic ability, thus implicating the type-I IFNs in modulating microglial phenotype and function (Moore et al., 2020). Considering the similarities between AD and PD, these findings support that type-I IFNs are key modulators of the chronic inflammatory process evident in neurodegenerative diseases and are potentially driving the disease progression through the actions of microglia.

## 8 Type-I IFNs in Parkinson's disease

There is increasing evidence for a deleterious role for the type-I IFNs in the progression of PD. Main et al. (2016) reported an elevated type-I IFN signature in post-mortem human PD brains, with increased mRNA expression of IFN-α, IFN-β, and IRF-7 compared to age-matched controls. Supporting a detrimental role for the type-I IFNs in PD, mice lacking the type-I IFN receptor (IFNAR1^−/−^) displayed attenuated neuroinflammation and microglial activation in response to the PD neurotoxin, MPTP. Importantly, these mice also displayed reduced dopaminergic neuronal death in the SNpc, compared to wildtype controls (Main et al., 2016). In support of the neuroprotective effects seen with type-I IFN signalling ablation, pharmacological inhibition of IFNAR1 using a monoclonal antibody, MAR-1, also conferred neuroprotection with a downregulation of inflammatory mediators (IFN-β, IRF-7 and IL-1β), reduced microglial activation in the SNpc, and increased dopaminergic cell survival (Main et al., 2016). Recently, it has been postulated that IRF-7 modulates microglial phenotypic switching towards a pro-inflammatory state (Tanaka et al., 2015) with this supported by a subsequent *in vitro* study by Main et al. (2017). They demonstrated that type-I IFNs produced by glial cells drive rotenone-induced cell death of co-cultured primary neurons (Main et al., 2017). Significantly, this response was attenuated in *IFNAR1*^*−/−*^ or MAR-1 pre-treated primary glia. Furthermore, a recent study identified transcription factors NFATc2 as key regulators of both the type-I IFN and microglial pro-inflammatory response. Specifically, the suppression of NFATc2 reduced IFN-β levels and phosphorylation of STAT1 in MPTP-treated BV2 cells, while the downregulation of NFATc2 rescued SH-SY5Y cells co-cultured with conditioned medium (Quan et al., 2024). Together, these findings highlight the crucial crosstalk between different cell types within the CNS with the type-I IFNs playing a pivotal role in modulating their responses to influence neuronal cell survival in disease settings.

STING, as an alternate inducer of type-I IFN signaling, has also been implicated in the neuroinflammatory response in PD. Genetic mutations in *PARK2*, an E3 ubiquitin ligase, and *PINK1,* a ubiquitin kinase, are associated with early-onset PD. Both Parkin and PINK1 are involved in mitophagy, the process of removing damaged mitochondria to prevent the buildup of mitochondrial stress. Importantly, a study showed that not only did mitochondrial stress lead to a STING-mediated type-I IFN response in *Parkin*^−/−^ and *PINK1*^−/−^ mice, STING removal and IFNAR1 blockage were able to rescue dopaminergic neuronal loss and attenuate inflammation (Sliter et al., 2018). This was in concordance with a subsequent study that showed STING-deficient mice were protected from dopaminergic neuronal loss in the α-syn PFF injection model, highlighting a role for the cGAS-STING and the downstream type-I IFN signalling pathway in driving the PD-associated neurodegeneration (Hinkle et al., 2022). Moreover, the cGAS-STING pathway is a key component in the senescence process, as the environmental stressors for the initiation of senescence converge through this signalling pathway (Huang et al., 2023). It was shown that deletions of cGAS and STING were able to diminish senescent phenotypes associated with oxidative stress and cell-cycle arrest (Gluck et al., 2017).

Moreover, type-I IFNs have been linked with other key cellular signalling pathways that are critical in maintaining cellular health. For instance, Wnt/β-catenin signalling is important in regulating neurogenesis and cell migration and has been shown to inhibit oxidative stress and inflammation in neurons (Ma and Hottiger, 2016). A study revealed that the protein β-catenin was essential for the activation of the cGAS-STING pathway in innate immunity, supporting an active interplay between the two signalling pathways (You et al., 2020). In addition, Wnt/β-catenin signalling has been shown to be downregulated by age-dependent inflammation (Marchetti et al., 2020). With an increased type-I IFN signature in an aging brain, the resultant dysregulation in Wnt/β-catenin signalling could also be a contributing factor to PD-related pathological changes, affecting the crosstalk between neurons and glial cells (L'Episcopo et al., 2018; Serafino and Cozzolino, 2023). Therefore, accumulating evidence is converging in the likely therapeutic benefits with targeting the type-I IFNs in PD due to their regulatory roles in not only the neuroinflammatory response but also other signalling pathways that synergistically exacerbate the neuronal environment.

Furthermore, targeting the type-I IFNs in PD could have beneficial effects for a number of non-motor symptoms. It has been shown that type-I IFNs play an important role in modulating the function of the intestinal epithelium (Katlinskaya et al., 2016). Indeed, the gut-brain axis has gained significant interest over the years, attributed to its suggested involvement in early PD pathogenesis. It is well known that PD patients often exhibit signs of gastrointestinal dysfunction including constipation, which commonly precede the onset of motor symptoms by years (Braak et al., 2003b; Devos et al., 2013). This is also supported by findings of intestinal inflammation in these patients, implicating a PD-related pathological process occurring in the periphery. Moreover, a study reported that gut microbiota influence PD symptoms and modulate motor deficits, microglial inflammation, and α-syn pathology in a mouse model of PD (Sampson et al., 2016). It seems likely that by targeting the type-I IFN signalling, inflammation in both the CNS and the gut can be attenuated to some extent, which may lead to better regulation. Meanwhile, aberrant type-I IFN signalling has been correlated with cognitive impairments in other age-related neurodegenerative diseases. It was identified that Aβ exposure in an AD murine model elicited robust type-I IFN production in microglia, with the blockade of the receptor IFNAR being able to rescue both memory and synaptic deficits (Roy et al., 2022). Since PD is increasingly recognised to be a multisystem disorder, the varying degrees of cognitive deficits in PD cases could serve additional insights into how type-I IFN-driven neuroinflammation contributes to cognitive impairments.

## 9 Challenges in targeting the type-I IFN signalling

Developing treatment strategies for PD is a challenging task due to the nature of the disease. Since clinical symptoms, disease severity, rate of progression can vary greatly among patients, there is no unified measure or disease biomarker to effectively segregate different disease stages. In addition, this review has illustrated the complexity of the overarching cellular signalling pathways involving the type-I IFNs and their key roles in the innate immunity. Thus, the use of type-I IFN-related mediators as therapeutic targets in PD calls for caution and needs to be carefully evaluated to avoid issues with compromised immune defence. It is also worth noting that the set of type-I IFN-regulated genes can be alternatively induced through other pathways such as endosomal toll-like receptors and cytosolic pattern recognition receptors (Paredes and Niewold, 2020). This can further complicate the readout of type-I IFN signature in PD as it will be difficult to measure their expression levels inside the brain.

Despite the anticipated challenges, the notion of targeting type-I IFN signalling has been extensively investigated in other chronic inflammatory conditions and shown promising results. Specifically, type-I IFN antagonists have been actively explored for their clinical use in treating Systemic Lupus Erythematosus (SLE), an autoimmune condition that causes extensive inflammation and tissue damage (Paredes and Niewold, 2020). Numerous clinical trials for assessing drug candidates including anti-IFN-α antibodies and anti-IFNAR antibodies have been carried out in SLE patients (Maurer et al., 2015; Merrill et al., 2011). To date, Anifrolumab, an IFNAR receptor blocker, has been shown to neutralize the IFN signature and with the potential for clinical use (Riggs et al., 2018). It seems that there could exist a distinct difference between the ligands of the type-I IFN signalling, with IFN-α exhibiting a likely pathogenic role whilst IFN-β being mostly physiological. Whilst both classified as type-I IFNs, it has been established that 13 subtypes of IFN-α exist, however there is only one isoform of IFN-β. Despite signalling through the same receptor, IFN-α and IFN-β can differ significantly in antiviral potency, receptor binding affinity, and immunomodulatory functions (Cook et al., 2019; Zhang et al., 2008). In the context of PD, mice lacking IFN-β exhibit age-dependent motor deficits and cognitive impairment, similar to traits seen in PD-like dementia (Ejlerskov et al., 2015, 2020). Additionally, increased apoptotic neurons and reduced neurite network formation were observed in *IFNβ*^*−/−*^ primary cerebellar granular neurons and cortical neurons (Ejlerskov et al., 2015). The lack of IFN-β was shown to cause autophagy defects, which led to inefficient clearance of senescent mitochondria and the accumulation of α-syn in a PD model. Nevertheless, given that the anti-IFN antibodies are too big a size to pass the blood brain barrier, clinical use of such molecules seems unlikely for PD management.

Recent development of small inhibitors that target the cGAS-STING pathway have unraveled novel avenues for PD treatment. Whilst the therapeutic targets are located upstream of the type-I IFN signalling pathway, the beneficial outcomes have suggested successful attenuation of the type-I IFN-driven neuroinflammation and the improved neuronal survival in pre-clinical studies (Hinkle et al., 2022). Importantly, a number of cGAS inhibitors and STING inhibitors have demonstrated brain permeability as a hurdle requirement for future PD treatment, suggesting that these therapeutic agents could hold promise in reaching the neuronal environment and limit the type-I IFN-mediated inflammation in PD (Decout et al., 2021). Given the role of cGAS and STING in the cell senescence and aging process, it is likely that targeting this signalling could yield a synergistic improvement in age-related neurodegenerative condition like PD, nevertheless this requires further characterisation of the drug molecules regarding the safety and pharmacokinetic profiles (Huang et al., 2023; Decout et al., 2021).

## 10 Conclusions

It is well established that neuroinflammation is a key pathological feature of neurodegenerative diseases such as PD and AD. Microglia are the major immune cell type involved in initiating and maintaining the neuroinflammatory response through the release of a multitude of inflammatory mediators that can act on multiple cell types within the CNS. Such signals propagate and activate other cells in the CNS and periphery to further amplify the pro-inflammatory microenvironment, resulting in chronic inflammation. In PD, it is postulated that neuroinflammation induces oxidative stress and cellular damage, affecting dopaminergic neurons in the SN and prompting them to degenerate. Indeed, overactivation of the inflammatory pathways has been linked with age-related CNS pathologies and more recently, an increasing body of evidence supports a critical role for type-I IFNs in driving this neuroinflammatory response (Taylor et al., 2018; Baruch et al., 2014; Hinkle et al., 2022). The generation and use of gene knockout murine models, such as the *STING*^*−/−*^ and *IFNAR1*^−/−^ mice, have allowed valuable insights into the role of this pathway in PD pathogenesis. However, neuroinflammation is an extremely complex process and indeed there is an increasing interest in the contribution of the peripheral immune response, specifically the gut-brain axis, in PD. With the recent development of cGAS-STING inhibitors, there could be promising therapeutic strategies that can limit the neuroinflammatory changes driven by type-I IFN signalling and achieve neuroprotective effects. Therefore, there is pressing need for a greater understanding of the disease mechanisms regarding how type-I IFNs exacerbate PD pathologies, to validate and develop novel therapeutic avenues that can truly slow PD progression.

## CRediT authorship contribution statement

**Shuyan Chen:** Writing – original draft. **Peter J. Crack:** Writing – review & editing, Resources, Funding acquisition, Conceptualization. **Juliet M. Taylor:** Writing – review & editing.

## Funding

Parkinson's Foundation Impact Award (JMT) and Melbourne Research Scholarship (SC).

## Declaration of competing interest

The authors declare no competing interests.

## Data Availability

No data was used for the research described in the article.

## List of abbreviations

**AD**: Alzheimer's disease
**ATP**: adenosine triphosphate
**BBB**: blood-brain barrier
**cGAS-STING**: cyclic GMP-AMP synthase/stimulator of interferon genes
**CNS**: Central nervous system
**DA**: Dopamine/dopaminergic
**DAM**: Disease-associated microglia
**DAMP**: Damage-associated molecular pattern
**DNA**: Deoxyribonucleic acid
**ER**: endoplasmic reticulum
**GDNF**: glial cell line derived neurotrophic factor
**GWAS**: Genome-wide association studies
**HLA**: Human leukocyte antigens
**IFN**: Interferon
**IFNAR1**: Interferon receptor alpha-1
**IL-1β**: Interleukin-1β
**IL-6**: Interleukin-6
**IRF-7**: Interferon regulatory factor-7
**JAK-STAT**: Janus kinase/signal transducers and activators of transcription
**LB**: Lewy body
**MAPK**: Mitogen-activated protein kinase
**MHC-II**: Major histocompatibility complex class II
**MPTP**: 1-methyl-4-phenyl-1,2,3,6-tetrahydropyridine
**MS**: Multiple sclerosis
**NFkB**: nuclear factor kappa B
**PD**: Parkinson's disease
**PET**: Positron emission tomography
**RNA**: ribonucleic acid
**SLE**: Systemic lupus erythematosus
**SNpc**: substantia nigra pars compacta
**TNF-α**: Tumour necrosis factor-α
**TLR**: Toll-like receptor
**TYK**: Tyrosine kinase
**α-syn PFF**: Alpha-synuclein pre-formed fibril
